# Shiftwork sleep disorder and associated factors among nurses working at public hospitals in Harari Regional state and Dire Dawa Administration, Eastern Ethiopia: a cross-sectional study

**DOI:** 10.1186/s12912-023-01257-1

**Published:** 2023-04-13

**Authors:** Henok Abate, Shiferaw Letta, Teshager Worku, Dejene Tesfaye, Eldana Amare, Ayalnesh Mechal

**Affiliations:** 1 School of Nursing and Midwifery, College of Health Science and Medicine, Jigjiga University, Jigjiga, Ethiopia; 2 School of Nursing and Midwifery, College of Health and Medical Sciences, Haramaya University, Harar, Ethiopia; 3 Comprehensive Nursing Department, College of Medicine and Health Sciences, Wachemo University, Hosanna, Ethiopia

**Keywords:** Shift-work sleep disorder, Nurses, Public hospitals, Eastern Ethiopia

## Abstract

**Background:**

Shiftwork sleep disorder is one of the most common health-related effects of Shiftwork, particularly among healthcare workers. It is a chronic condition that is directly related to a person’s work schedule. In Ethiopia, although a mental health strategy is in place, little attention is given to studies that focus on shiftwork sleep disorders among nurses. This study aimed to determine the magnitude of shiftwork sleep disorder and associated factors among nurses working at public hospitals in Harari Regional State and the Dire Dawa Administration.

**Methods:**

Institutional based cross-sectional study was conducted from June 1–30, 2021 among 392 nurses selected by a simple random sampling technique. A structured interviewer-guided self-administered questionnaire was used for data collection. The International Classification of Sleep Disorders 3rd edition (ICSD-3), Bargen Insomnia Scale (BIS) and Epworth Sleepiness Scale were used to assess shift-work sleep disorder. The data were entered into EpiData and exported to SPSS for analysis. Bivariable logistic regression was used to see the association between the outcome and the explanatory variables. Bivariate and Multivariate analyses were performed, and AOR with 95% CI was used to measure the strength of the association. Those variables with a p-values of < 0.05 were considered as statistically significant.

**Results:**

In this study, the magnitude of shiftwork sleep disorder among nurses was 30.4% (95% CI: 25.4–34.5). Being female (AOR = 2.4, 95% CI: 1.3, 4.2), working an average number of nights > 11 per month in the last 12 months (AOR = 2.5, 95% CI: 1.3, 3.8), and khat use in the last 12 months (AOR = 4.9, 95% CI: 2.9, 8.7) were significantly associated with the shiftwork sleep disorder.

**Conclusions:**

The study revealed that about one-third of the nurses had a shiftwork sleep disorder implying a high burden of the problem among nurses in the study setting, which endangers nurses, patients, and the healthcare system. Being female, working an average number of nights > 11 per month in the last 12 months, and khat use showed statistically significantly associated with the shiftwork sleep disorder. Early detection of shiftwork sleep disorder, having a policy on khat use and considering rest/recovery while scheduling work time should be addressed to prevent shiftwork sleep disorder.

## Introduction

Shiftwork refers to a broad range of non-standard work schedules, ranging from occasional on-call overnight duty to rotating timetables, to stable, permanent night work, and programs demanding an early awakening from nocturnal sleep [1]. It includes fixed early morning, evening, and night work, as well as roster and rotating three-shift work [2]. Evidence showed that there are various shift schedules. The medical nurses usually provide a 24-hours service, on rotating schedules while the eight‐hour and 12‐hour shifts are the most common scheduling systems [3]. The National Sleep Foundation defined shift-work sleep disorder (SWSD) as a chronic condition that is directly related to a person’s work schedule. SWSD is considered a ‘circadian rhythm sleep disorder’ (CSRD) by the International Classification of Sleep Disorders 3rd edition (ICSD-3) [4, 5]. Circadian rhythm sleep disorder is a sleep pattern disorder caused by changes in biological hours in humans [6]. Shiftwork sleep disorder is characterized by insomnia or sleepiness that occurs in association with Shiftwork [7].

Studies done in developed and developing countries showed that a significant number of nurses suffer from shiftwork sleep disorders [8, 9] Results of various studies indicated that a huge number of nurses engaged in Shiftwork were found to be affected by shiftwork-related sleep disorders. According to an e-mail-based cross-sectional study done in Newcastle upon Tyne, North East England, the prevalence of poor sleep quality (PSQI > 5) in shift-working nurses was 78% [10]. In a cross-sectional study done in seven central Italian hospitals, 52.1% had suffered from poor sleep quality [11], another study done among nurses working in nocturnal and diurnal shifts in a public hospital, the prevalence of bad sleep reported was 68.3% [12]. Similarly, in a study done in Sweden, night shift insomnia was 67% in three-shift rotation work and 41.7% in nurses with permanent night work [13].

In Asian countries, cross-sectional studies conducted among nurses revealed that the prevalence of SWSD was 24.4% in Japan [14], 32.2% in South Korea [15], 75.0% had a significant sleep problem, and 39.7% of subjects had inadequate sleep stability in Northern Taiwan [16], and 24.6% of the nurses had experienced at least one of the five insomnia symptoms in Thailand [17].

The findings of a cross-sectional study conducted on nurses working in hospitals in the south of Iran showed that 56**%** of them had signs of insomnia, and of all the participants, 78.5% were sleepy, 16.5% were very sleepy, and 5% were severely sleepy [18]. Studies in some African countries reported that SWSD affected 43.2% and 20.8% of nurses in Nigeria and Ghana respectively [19, 20]. A comparative cross-sectional study conducted in Egypt, 73% of nurses working in a shift had poor subjective sleep quality [21].

Similarly, a study in Ethiopia found that about a quarter (25.6%) of the nurses experienced SWSD [22]. Different studies reported factors like age, sex, Shiftwork -related factors [23–25], substances use (caffeine, alcohol, cigarettes and others) [26–28] and using different forms of sleep aid [24] were associated with SWSD among nurses. One study conducted among health professionals revealed that khat chewing was significantly associated with SWSD [29].

The shift-work sleep disorder can be associated with decreased attention and deficit in cognitive functioning which in turn decreased performance that may contribute to a higher propensity for mistakes/near misses/accidents. It is also associated with an increased prevalence of medical disorders such as cancer, decreased quality of life, and increased risk of mood disorders (anxiety and depression) [30–33]. Studies indicated that poor sleep quality and insufficient sleep contribute to psycho-physiological health problems such as fatigue, emotional disturbance, and cardiovascular disorders [34, 35]. It also affects decision-making ability and an increased propensity for accidents both on and off the job and results in decreased efficiency and productivity. Moreover, it increases the risk of making medical errors, compromised healthcare quality, patient safety and occupational injuries [36, 37]. SWSD significantly increased workers’ absenteeism which harms employers economically [30]. Regarding the management of SWSD, designed bright‐light exposure, taking naps [38, 39], shift schedule rearrangement [38] and taking drugs like Armodafinil are used to treat and prevent SWSD [40].

Nurses use or abuse different types of substances, like non-prescribed medications, alcohol, khat or cigarette to alleviate the stress associated with shift work [22, 41]. Shiftwork by itself was associated with nurses’ use/abuse of substances [42].

In Ethiopia, there are no policies designed to prevent shiftwork-related sleep disorders. There is a paucity of information regarding the extent of SWSD among nurses, particularly in Eastern Ethiopia. This study tried to explore the association of SWSD with depression, anxiety, and stress that were not addressed in the previous studies done in the country. Therefore, the findings from this study could generate local evidence that would inform policymakers & local planners to design cost-effective strategies that reduce adverse health outcomes of SWSD among nurses in Ethiopia.

## Methods and materials

### Study setting and design

An institution-based cross-sectional study was conducted from June 1–30, 2021 at four public hospitals in Harari Regional State and Dire Dawa Administration, Eastern Ethiopia. Harar is the capital city of the Harari Regional State. In the city, there were two public hospitals, Hiwot Fana Specialized University Hospital (HFSUH) and Jugal General Hospital (JGH). The health management information system of the hospitals reported that there were 256 nurses in HFSUH and 103 nurses in JGH. In Dire Dawa Administration, there were two public hospitals namely, Dilchora Referral Hospital (DRH) and Sabian General Hospital (SGH). According to the hospitals’ health management information system report, there were 163 nurses in DRH and 93 nurses in SGH.

### Study population and sampling procedure

All nurses who were working in the four public hospitals in Harari Regional State and Dire Dawa Administration. Those who had work experience of greater than 6 months were included, whereas all nurses with diagnoses of any type of sleep disorder and nurses working in a standard non-shift day program were excluded from the study. The sample size was calculated using the single population proportion formula for the first objective. Accordingly, the estimated sample size was 323 including the 10% non-response rate. The sample size was also calculated for the factors associated with SWSD, and was determined by double population proportion formula using EpiInfo software, considering the following assumptions: 95% confidence level, 80% power, 1:1ratios, exposed vs. non-exposed were 35.3%, and 21.4%, AOR: 3.1; from a previous study done in Addis Ababa [22] with 10% non-response rate., In light of this, 392 Nurses were included in this study as the final sample. The total samples were distributed proportionately to each hospital from a pool of 615 nurses in the selected public hospitals. Finally, study participants were selected using a simple random sampling technique with a computer-assisted random number generator.

### Data collection procedure and data quality control

Data were collected by interviewer-guided self-administered questionnaires prepared in the English language. Data were collected by eight BSc and four diploma nurses and supervised by four BSc nurses. A two-day training was given to the data collectors and the supervisors on the objective of the study, data collection procedures and ethical aspects of the entire data collection process. The questionnaire was pretested among 10% of the total sample size before the actual data collection at Bisidimo General Hospital, Eastern Hararghe Zone, Oromiya Reginal State.

### Study variables and measurements

The questionnaire comprised, socio-demographic information, shiftwork-related characteristics, substance and sleep aid medication use, and the existence of clinical and mental health-related characteristics such as depression, anxiety, and stress symptoms. A shiftwork sleep disorder was assessed by using the International Classification of Sleep Disorders-third (ICSD-3), which helps to determine sleepiness and insomnia [43]. The ICSD-3 has three items with Yes or No responses: (1) experiencing difficulties with sleeping or excessive sleepiness (yes/no); (2) Are you having trouble sleeping due to a work schedule that requires you to work at times when you would normally sleep? ; (3) Has your sleep or sleepiness problem been persistent for at least three months? To qualify for a shiftwork sleep disorder, respondents had to answer ‘yes’ to all three questions [44]. A sleepiness and insomnia assessment tools were included. The Bargen Insomnia Scale (BIS) is a self-administered insomnia scale with symptom-related questions based on the American Psychiatric Association Diagnostic and Statistical Manual of Mental Disorders-IV-text revision (DSM-IV-TR) [45]. Using an 8-point scale, six items indicate how many days per week a particular symptom occurs (0–7 days, total scores of 0 to 42). Scoring 3 or more on at least one of items 1–4, and 3 or more on at least one of items 5 and 6, of the BIS, was considered as having insomnia. The scale has been validated and has Cronbach’s alpha values ranging from 0.79 to 0.87 [46]. In the present study, Cronbach’s alpha for the BIS was 0.86. Based on the Epworth Sleepiness Scale (ESS), respondents estimated the likelihood that they would fall asleep in each of eight situations, ranging from 0 = never would fall asleep to 3 = highly likely to fall asleep. Scores can range from 0 to 24. In clinical studies, the ESS score (clinical cut-off 11) can distinguish between patients with sleep disorders and healthy participants. The tool has good reliability, including in Ethiopia (Cronbach’s alpha = 0.75) [47]. Based on the data in the current study, Cronbach’s alpha was 0.83 for the ESS. In this study, SWSD was defined as fulfilling the criteria for ICSD-3 and BIS and/or ESS. A depression, anxiety, and stress scale (DASS-21) consist of 21 items. The three subscales contain, 7 items for each of depression, anxiety, and stress. The rating scale is as follows: 0 = never applied to me, 1 = applied to me sometimes, 2 = applied to me often, and 3 = applied to me almost always. Those who scored greater than 7, 9 and 15, indicate anxiety, depression and Stress on the subscales [48] respectively.

### Data analysis

Before data entry, the questionnaires were checked for completeness, consistency and clarity. Coded data were entered using EpiData v-3.1 software [49] and exported to SPSS (v-26, Armonk, NY: IBM Corp) for cleaning and analysis. Descriptive statistics were calculated for all variables to summarize the data. The findings of the study were presented using tables and graphs. Those variables with a p-value **< 0.25** in the bivariable logistic regression analysis were taken to the multivariable logistic regression to identify factors associated with SWSD and a significant statistical association was declared at a p-value of **< 0.05.** Adjusted odds ratios with corresponding 95% confidence intervals were used to show the strength of the association. The model fitness was checked by the Hosmer-Lemeshow model fitness test and its p-value was 0.624. The independent variables did not exhibit collinearity or multi-collinearity.

## Results

### Socio-demographic characteristics

Out of 392 randomly selected nurses, 369 nurses participated in this study, yielding a response rate of 94%. The mean age of the study participants was 31.25 (SD ± 6.97) years and ranges from 20 to 60 years. More than half (n = 200, 54.2%) of them were male and about 197 (53.7%) were married. Most of the study participants (n = 315, 85.4%) were BSc degree holders, 157 (42.5%) had work experience of fewer than 5 years and close to one-third (n = 110, 29.8%) worked in a medical-surgical unit (Table 1).

**Table 1:** Socio-demographic characteristics of the nurses working at public hospitals in Harari Regional State and Dire Dawa administration, Eastern Ethiopia, 2021(n = 369)

Variables	Categories	Frequency	Percentage
Age	20–29	185	50.1
	30–39	134	36.3
	≥ 40	50	13.6
Sex	Male	200	54.2
	Female	169	45.8
Marital status	Single	151	40.9
	Married	198	53.7
	Others*	20	5.4
Educational status	Diploma	35	9.5
	BSc degree	315	85.4
	Master’s degree	19	5.1
Department	Medical & Surgical	110	29.8
	Pediatrics	65	17.6
	ICU & Operation room	71	19.4
	Emergency	63	17.1
	Other**	60	16.3
Average monthly income	< 3934 ETB	13	3.5
	3935 to 6193 ETB	210	56.9
	> 6193 ETB	146	39.6
Work experience	< 5years	157	42.5
	5-10years	141	38.2
	> 10years	71	19.2

other*= living together, divorced, and separated **other: gynecology, obstetrics, neonatal ICU, orthopedics

### Shiftwork and sleep medication use related characteristics

About two-thirds of the study participants (n = 258, 69.9%) were working in a two-Shiftwork program. Two hundred twenty-four (60.7%) of them were working 8–11 night shifts per month and about two-thirds, (n = 256, 69.4%) of nurses took a nap during their night shift. In this study, 37(10%) nurses reported that they had used prescribed sleep medication and 19(5.1%) reported they have used non-prescribed injectable sleep medications in the last 12 months (Table 2).

**Table 2:** Other Shiftwork -related characteristics of nurses working at public hospitals in Harari Regional State and Dire Dawa administration, Eastern Ethiopia, 2021(n = 369)

Variables	Category	Frequency	Percentage
Frequency of rotation per day	Two shifts	258	69.9
	Three shifts	111	30.1
Length of night shift time	8 h	43	11.7
	12 h	80	21.7
	14 h	246	66.7
Number of night shifts per month	< 7 days	37	10
	8–11 days	224	60.7
	> 11 days	108	29.3
Taking a nap	Yes	256	69.4
	No	113	30.6
Used prescribed sleep medication	Yes	37	10
	No	332	90
Used non-prescribed sleep medication	Yes	6	1.6
	No	363	98.4
Used non-prescribed injectable sleep medication	Yes	19	5.1
	No	350	94.9

### Mental and clinical related characteristics

The study revealed that 57 nurses (15.4%) reported having a diagnosed chronic illness, and of these, 23 nurses (6.2%) had diabetes mellitus. Regarding mental health, 16 (4.3%) reported having a history of diagnosed mental illness in the past, while 13 (3.5%) had a family history of mental illness. Similarly, depression, anxiety, and stress were present in 100 (27.1%), 109 (29.5%), and 61 (16.5%) of the study participants, respectively. (Table 3).

**Table 3:** Clinical characteristics (variables) of nurses working at public hospitals in Harari Regional State and Dire Dawa Administration, Eastern Ethiopia, 2021 (n = 369)

Variables	Category	Frequency	Percentage
History of chronic medical illness?	Yes	57	15.44
	No	312	84.56
Types of chronic medical illness	Hypertension	15	4.1
	DM	21	5.7
	HIV/AIDS	7	1.9
	Asthma	8	2.2
	*Other	8	2.2
History of mental illness in the past	Yes	16	4.3
	No	353	95.7
Types of the mental disorders in the past	Depressive disorder	6	1.6
	Psychotic disorder	4	1.1
	‡Other	7	0.3
Family history of mental illness	Yes	13	3.5
	No	356	96.5
Types of mental disorders in the Family history	Depressive disorder	5	1.4
	Anxiety disorder	4	1.1
	‡Other	4	1.1
Depression	Yes	100	27.1
	No	269	72.9
Anxiety	Yes	109	29.5
	No	260	70.5
Stress	Yes	61	16.5
	No	308	83.5

*Other: Cardiac case, Tuberculosis, and cancer. ‡Other: Psychosomatic disorder, Anxiety disorder, bipolar disorder

### Substance-related characteristics

Almost half of the study participants, 182 (49.3%) had ever used Khat in their lifetime, while 154(41.7%) had used Khat in the last 12 months, of the total, 61 (16.5%) had ever used tobacco products, and 300 (81.3%) had used caffeine in the previous 12 months. (Table 4).

**Table 4:** Substance use distribution among nurses working at public hospitals of Harari Regional State and Dire Dawa Administration, Eastern Ethiopia, 2021(n = 369)

Variables	Category	Frequency	percentage
Ever used khat in life	Yes	182	49.3
	No	187	50.7
Used khat in the last the 12 months	Yes	154	41.7
	No	215	58.3
Ever drank alcohol in life	Yes	106	28.7
	No	263	71.3
Drank alcohol in last the 12 months	Yes	64	17.3
	No	305	82.7
Ever used tobacco products in life	Yes	63	17.1
	No	306	82.9
Used tobacco products in last the 12 months	Yes	24	6.5
	No	345	93.5
Used caffeinated drink in the last the 12 months	Yes	300	81.3
	No	69	18.7

### Shiftwork sleep disorder among nurses

**Fig. 1:**
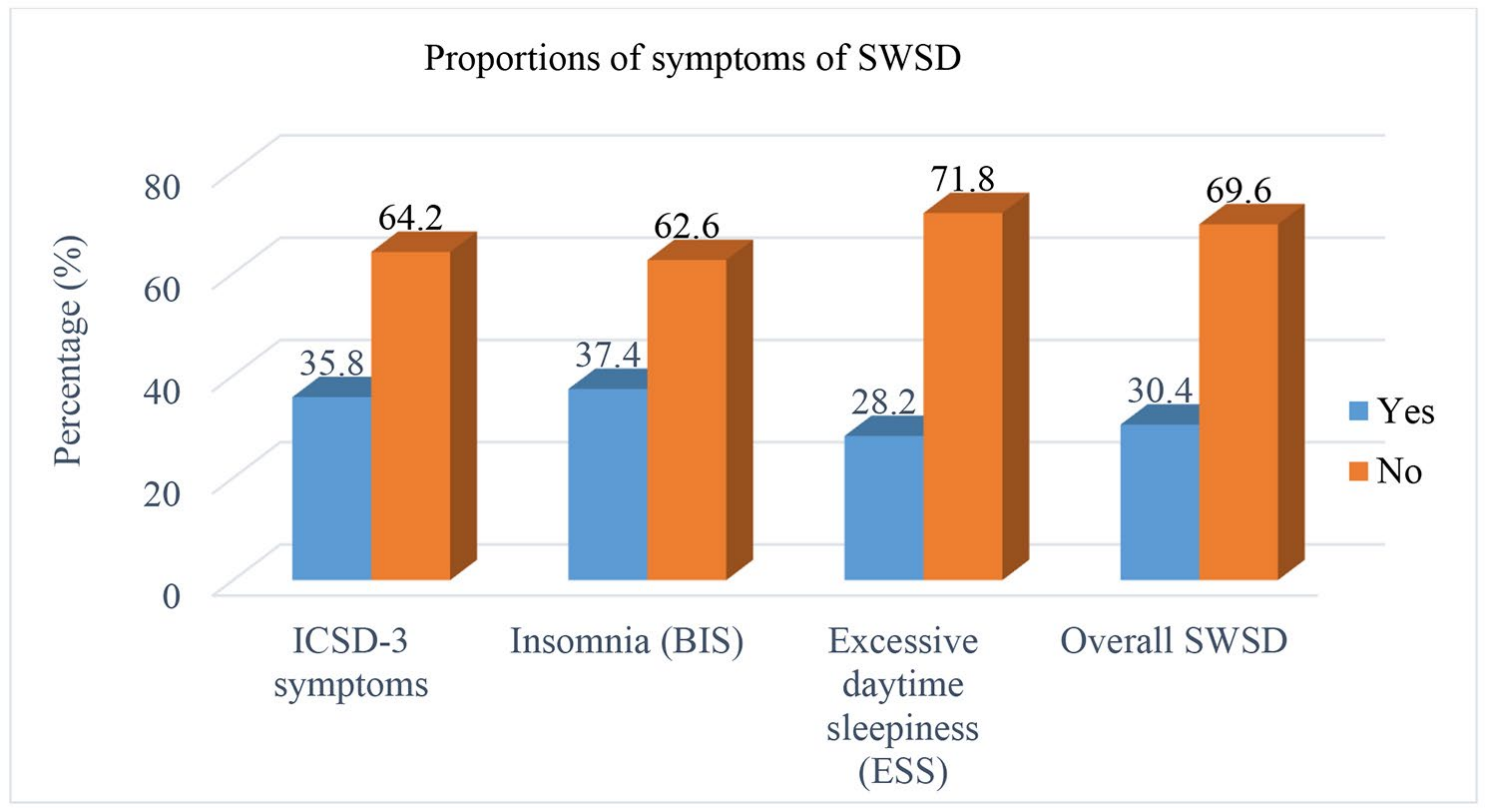
SWSD disorder and its symptoms among nurses working at public hospitals of Harari Regional State and Dire Dawa Administration, Eastern Ethiopia, 2021

Overall, 112(30.4%) (95% CI: 25.4–34.5) of the nurses had shiftwork sleep disorder. In this study, 132 (35.8%) full filled the criteria of ICSD-3 (participating nurses who said yes to all three questions of ICSD-3), 104(28.2%) had excessive daytime sleepiness and 138(37.4%) of the nurses had insomnia (Fig. 1).

### Factors associated with SWSD

In bivariable logistic regression, age, sex, work experience, nap-taking during the night shift, the average number of nights per month in the last 12 months, use of prescribed sleep medication in the last 12 months, chronic medical illness, depression, anxiety, stress, ever use of tobacco, use of tobacco in the last 12 months, used khat in the last 12 months were associated with SWSD, whereas, being female, working more than 11 nights per month in the last 12 months on average and being a user of khat in the last 12 months were the factors that had statistically significant association with SWSD (Table 5).

**Table 5:** Factors associated with SWSD among nurses working at public hospitals in Harari Regional State and Dire Dawa Administration, Eastern Ethiopia, 2021(n = 369)

Variable	Category	SWSD		Crude OR(95%CI)	Adjusted OR(95%CI)
		Yes (%)	No (%)		
Age	20–29 year	47	138	1	1
	30–39 year	40	94	1.25(0.76–2.05)	1.28(0.63–2.60)
	≥ 40 year	25	25	2.94(1.54–5.6)	1.74(0.59–5.18)
Sex	Male	50	150	1	1
	Female	62	107	1.74 (1.11–2.72)	2.37(1.34–4.18)**
Work experience	< 5 year	38	119	1	1
	5–10 year	43	98	1.37 (0.82–2.23)	1.18(0.58–2.39)
	> 10 year	31	40	2.43 (1.34–4.40)	0.89(0.32–2.45)
Nap during night Shiftwork time	Yes	71	185	1	1
	No	41	72	1.48(0.93–2.38)	1.36(0.79–2.37)
Average number of nights per month in the last 1 year	≤ 11	58	188	1	1
	>11	54	71	2.47(1.54–3.87)	2.27(1.34–3.84)**
Used prescribed sleep medication in the last 1 year	Yes	21	16	3.48(1.74–6.96)	2.18(0.92–5.19)
	No	91	241	1	1
Chronic medical illness	Yes	26	31	2.20 (1.24–3.93)	1.05(0.42–2.62)
	No	86	226	1	1
Depression	Yes	38	62	1.62(0.99–2.62)	0.74(0.28–1.95)
	No	74	195	1	1
anxiety	Yes	41	68	1.61(0.99–2.58)	0.88(0.37–2.10)
	No	71	189	1	1
stress	Yes	25	36	1.76 (1.00-3.11)	0.77(0.32–1.86)
	No	87	221	1	1
Ever used tobacco	Yes	27	34	2.08(1.19–3.66)	1.21(0.59–2.47)
	No	85	223	1	1
Used tobacco in the last 12 months	Yes	21	24	2.14(1.19–4.22)	1.33(0.63–2.81)
	No	91	233		1
Used khat in last 12 month	Yes	75	78	4.65(2.89–7.48)	4.99(2.88–8.65)***
	No	37	179	1	1

** = p-value < 0.05 & *** = p-value < 0.01.

Nurses, who were female were 2.4 times more likely to develop SWSD as compared to their male counterparts (AOR: 2.4; 95%; CI: 1.3–4.2). The odds of SWSD were 2.3 times higher among nurses who worked more than 11 nights per month in the last 12 months on average than those nurses who worked less than 11 nights per month in the last 12 months on average (AOR: 2.3; 95%; CI: 1.3–3.8). The possibility of SWSD was increased by nearly about five folds among the nurses who used khat in the last 12 months compared to the nurses who did not use khat in the last 12 months (AOR: 50, 95%; CI: 2.9–8.7) (Table 5).

## Discussion

In the current study, the magnitude of SWSD was 30.4% (95%; CI: 25.4–34.5). Being female, working more than 11-night shifts on average per month in the last 12 months and nurses who consumed khat in the last 12 months were significantly associated with SWSD.

According to this study, nearly one in every three nurses suffered from a shiftwork sleep disorder that adversely affects the health of nurses, and patient’s safety, satisfaction, and rendered care quality. Empirical evidence indicated that circadian rhythm sleep disorders, insomnia, and excessive sleepiness are widespread in night-shift workers that are associated with substantial morbidity including accidents and absenteeism [30]. The result of this study was in line with a study conducted in Addis Ababa, Ethiopia [22]. However, it was found higher than in studies carried out in Japan [14] and Ghana [50]. The possible justifications for the observed discrepancy between the current study to the study done in Japan [14] might be due to the tool difference. A study in Japan used only the three questions of ICSD-3 criteria for assessment of SWSD and included all nurses working in two university hospitals in the Tokyo metropolitan while this study was a multicenter and the study participants were recruited randomly.

In the Ghanaian study [50], shiftwork sleep disorder was assessed with a tool which assessed the average amount of hours each nurse has for sleeping, but in our study SWSD was determined by ICSD-3, Insomnia and Excessive daytime sleepiness combined together, in addition to this sample size used were also lower than the sample size of the current study which might have caused the noted discrepancy. In addition to the above possible reasons, also the effect of khat might be the other reason which caused the discrepancy, as it is known that khat is the most commonly consumed substance in eastern parts of Ethiopia, which was significantly associated with sleep problems [51, 52]. On the other hand, compared to studies in Nigeria ( 43.2%) [23], Egypt [21], and England [10], the finding of the current study was lower. The possible justification for the discrepancy was the difference in study design and sample size. A the study done in Nigeria [23] used a case control study design with a smaller sample size and the current study employed a cross-sectional study design with a relatively larger sample of nurses. There was also variation in tools used for assessing SWSD. Studies were done in England and Egypt used Pittsburgh Sleep Quality Index (PSQI) while the current study determined SWSD by ICSD-3 criteria, BIS and ESS.

Being female appeared to be associated significantly with SWSD, which was in line with the systematic review conducted in 2011[25] and a community cohort study in Toronto, Canada, [53]. The possible reason for this association might be that there is a biological difference between males and females that females have common changes in hormonal levels; that may impact the sleep. Gonadal hormone cycles affect the sleep patterns of women during the menstrual cycle and menopause. This can result in more insomnia and frequent waking up during the sleep cycle [54].

In the current study, working more than 11-night shifts per month on average in the last 12 months showed an association with SWSD; this finding was consistent with the study done in Addis Ababa, Ethiopia [22] and South Korea[55] which assessed shiftwork tolerance among rotating shiftwork nurses. One possible explanation is that having less off-time leads to less sleep duration, which may result in shiftwork type circadian sleep disorder. While, night work does not necessarily limit nurses’ time to rest, a frequent night shift limits nurses’ opportunity for sleep and may cause them to spend a lot of time on non-work activities in between Nights.

Consuming khat in the last 12 months was positively associated with SWSD; this association might happen due to Cathinone (alkaloids found in khat leave which have stimulant activity similar to amphetamines). Using khat reduces dopamine re-uptake and activates dopaminergic pathways involved in the regulation of sleep. After the khat session, the user usually experiences a depressed mood, irritability, anorexia, and difficulty sleeping [56] and this might lead to circadian rhythm type sleep disorder or SWSD.

### Strengths and limitations of the study

This study included nurses in different types of hospitals like teaching hospitals, specialized hospitals, and general hospitals, and it was a multicenter study which generates vital evidence. The study used the standard and validated tools which facilitated approximating the burden of SWSD.

Though several efforts were made, this study had its limitations. Those nurses diagnosed with SWSD could also be diagnosed with some other forms of sleep disorders. Yet, some other nurses might have been transferred to daily shiftwork schedules Asking nurses who were expected to have knowledge about the adverse effects of substance use might introduce the risk of social desirability bias. There was also the risk of recall bias, and no measure was taken to minimize it. The nature of a cross-sectional study design was another major limitation since it cannot show temporal relationships between the dependent and the independent variables.

## Conclusion

About one-third of the nurses working at public hospitals in Harari Reginal State and Dire Dawa Administration, Eastern Ethiopia had SWSD. Being female, working an average number of nights > 11 per month in the last 12 months, and khat use showed a positive statistically significant association with the shiftwork sleep disorder. The prevention of shiftwork sleep disorder should focus on early detection, having a policy on khat use, and taking rest/recovery into account when scheduling work time. Moreover, longitudinal, and controlled trial studies will be necessary to highlight the effect of SWSD on the well-being of the nurses, patients’ safety, and the health care system.

## Data Availability

The data will be available from the corresponding author upon a reasonable request.

## Abbreviations

AOR: Adjusted Odds Ratio
BSc: Bachelor of Science
COR: Crude Odds Ratio
DASS-21: Depression, Anxiety and Stress Scale 21 item
DRH: Dilchora Referral Hospital
DSM-IV-TR: Diagnostic and Statistical Manual of Mental Disorders-IV-text revision
ESS: Epworth Sleepiness Scale
HFSUH: Hiwot Fana Specialized University Hospital
ICSD-3: International Classification of Sleep Disorders-Third Edition, General Hospital
JGH: Jugal General Hospital
MSc: Masters of science
OR: Odds Ratio
SGH: Sabian General Hospital
SWSD: Shift-Work Sleep Disorder
